# Absolute oral bioavailability and possible metabolic pathway of panduratin A from *Boesenbergia rotunda* extract in beagle dogs

**DOI:** 10.1080/13880209.2023.2190777

**Published:** 2023-03-30

**Authors:** Tussapon Boonyarattanasoonthorn, Teetat Kongratanapasert, Apisada Jiso, Pinnakarn Techapichetvanich, Nitra Nuengchamnong, Kittitach Supannapan, Anusak Kijtawornrat, Phisit Khemawoot

**Affiliations:** a Department of Physiology, Faculty of Veterinary Science, Chulalongkorn University, Bangkok, Thailand; b Chakri Naruebodindra Medical Institute, Faculty of Medicine Ramathibodi Hospital, Mahidol University, Samutprakarn, Thailand; c Program in Translational Medicine, Faculty of Medicine Ramathibodi Hospital, Mahidol University, Bangkok, Thailand; d Science Laboratory Centre, Faculty of Science, Naresuan University, Phitsanulok, Thailand; e Chao Phraya Abhaibhubejhr Hospital Foundation, Prachinburi, Thailand

**Keywords:** Pharmacokinetics, Zingiberaceae, fingerroot extract, Panduratin A

## Abstract

**Context:**

Attempts are ongoing to develop medications to fight against the COVID-19 pandemic. Our previous study revealed the *in vitro* anti-SARS-CoV-2 activity of fingerroot [*Boesenbergia rotunda* (L.) Mansf. (Zingiberaceae)] and its phytochemical, panduratin A.

**Objective:**

To investigate the pharmacokinetic profiles of panduratin A as a pure compound and in a fingerroot extract formulation in beagle dogs.

**Materials and methods:**

A total of 12 healthy dogs were randomly divided into three groups, a single dose of 1 mg/kg panduratin A by intravenous and multiple doses of 5 and 10 mg/kg panduratin A fingerroot extract formulation by oral administration for seven consecutive days. The plasma concentration of panduratin A was determined by LCMS.

**Results:**

The peak concentrations of a single dose of 5 and 10 mg/kg panduratin A fingerroot extract formulation were 12,416 ± 2,326 and 26,319 ± 8,221 µg/L, respectively. Increasing the oral dose of fingerroot extract formulation, equivalent to panduratin A 5–10 mg/kg, showed dose proportionality, with an approximately 2-fold increase in *C*_max_ and AUC. The absolute oral bioavailability of panduratin A in the fingerroot extract formulation was approximately 7–9%. The majority of panduratin A was biotransformed into several products *via* oxidation and glucuronidation, and predominantly excreted *via* the faecal route.

**Conclusion:**

The oral formulation of fingerroot extract was safe in beagle dogs, and increasing dose showed dose proportionality in terms of the systemic exposure of panduratin A. This information will support the phytopharmaceutical product development of fingerroot extract against the COVID-19 pandemic.

## Introduction

Coronavirus disease 2019 (COVID-19), caused by severe acute respiratory syndrome coronavirus 2 (SARS-CoV-2), was pronounced a pandemic on 11 March 2020 (WHO 2020). Globally, more than 0.5 million newly reported cases occurred every 7 d, over 600 million people were infected, and six million people died. Patients with COVID-19 exhibit a wide range of clinical symptoms, ranging from asymptomatic infection to critical illness. COVID-19 treatment guidelines recommend antivirals and dexamethasone or other systemic corticosteroids in hospitalized adult patients (NIH 2022). Remdesivir was the first approved antiviral drug for COVID-19 patients over 12 years old who require hospitalization (U.S. Food and Drug Administration [USFDA], 2020), although a cochrane review reported that remdesivir showed little or no effect on all-cause mortality at up to day 28 in hospitalized patients with SARS-CoV-2 infection (Ansems et al. 2021). Two other oral antiviral drugs, ritonavir-boosted nirmatrelvir and molnupiravir, were issued emergency use authorizations in December 2021 by the USFDA for the treatment of mild-to-moderate COVID-19 in adult patients (USFDA 2021a, 2021b). Although the EPIC-HR trial reported that ritonavir-boosted nirmatrelvir reduced the risk of hospitalization or death by 88% compared to placebo in non-hospitalized adults (USFDA 2021c), the limitation of ritonavir-boosted nirmatrelvir was the potential for drug–drug interactions from ritonavir (a strong CYP3A inhibitor); thus, this regimen may not be suitable for all patients. The use of molnupiravir is recommended when other antivirals cannot be given or are not available, due to only a 30% reduced rate of hospitalization or death by molnupiravir compared to placebo in the MOVe-OUT study (Jayk Bernal et al. 2021). These recent antivirals are not highly effective in treating hospitalized COVID-19 patients, and the discovery and development of drugs to treat this infection are important and urgent, to reduce infection and death.

Natural products are known to be the oldest source of drug discovery. Since the outbreak of COVID-19, there has been a dramatic increase in the use of medicinal plants and natural products for prevention and treatment (Boozari and Hosseinzadeh 2021). Thai medicinal plants and their components are promising sources of anti-SARS-CoV-2 agents. *Andrographis paniculata* (Burm.f.) Wall. ex Nees (Acanthaceae) is a Thai herb approved by the WHO for prophylaxis and symptomatic treatment of upper respiratory infections and acute diarrhea (World Health Organization [WHO] 2002). *A. paniculata* extract and andrographolide (its major compound) significantly inhibited SARS-CoV-2-infected Calu-3 cells, with an IC_50_ of 0.036 μg/mL and 0.034 μΜ, respectively (Sa-ngiamsuntorn et al. 2021), and the Royal Thai Government Gazette announced the use of *A. paniculata* capsules to treat asymptomatic COVID-19 patients in Thailand on 4 June 2021 (NDSDC 2021). In our previous study, 122 extracts and compounds from Thai medicinal plants were screened for antiviral activity against SARS-CoV-2-infected Vero E6 cells. *Boesenbergia rotunda* (L.) Mansf. (Zingiberaceae), also known as fingerroot extract, and its bioactive compound, panduratin A, exhibited anti-SARS-CoV-2 activity, with IC_50_ of 3.62 μg/mL and 0.81 μΜ, respectively, whereas the standard drug remdesivir showed an IC_50_ of 2.71 μΜ (Kanjanasirirat et al. 2020). In our preliminary study, administering fingerroot extract containing panduratin A 15–50 mg/kg/d for 7 d could reduce lung pathology in SARS-CoV-2 infected hamsters. According to interspecies scaling calculation (USFDA 2005), the appropriate dosing of panduratin A in dogs should be 5–10 mg/kg/d. Therefore, an oral formulation of fingerroot extract containing panduratin A 5–10 mg/kg/d was chosen to determine the systemic exposure, possible metabolic pathways, and the excretion route in beagle dogs. Because of the good antiviral activity of *B. rotunda* extract and panduratin A, this herb may be a possible candidate for COVID-19 drug development. Determining the efficacy, safety, toxicity and pharmacokinetic profiles of panduratin A from fingerroot extract in animal studies is crucial for further product development during this pandemic.

Panduratin A is a prenylated cyclohexenyl chalcone found in *B. rotunda* (fingerroot) and is reported to have antioxidant, anti-inflammatory, antibacterial, anticancer, antiviral and nephroprotective effects (Yun et al. 2003, 2006; Sohn et al. 2005; Cheenpracha et al. 2006; Kiat et al. 2006; Rukayadi et al. 2010; Thongnuanjan et al. 2021). The predicted physicochemical properties of panduratin A are two hydrogen bond donors, four hydrogen bond acceptors with a molecular weight of 406.5 g/mol, and XlogP = 7.08 (PubChem 2004). According to Lipinski’s rule of five, an orally active drug should have the following criteria: no more than 5 hydrogen bond donors, no more than 10 hydrogen bond acceptors, a molecular mass of fewer than 500 daltons, and a logP that does not exceed 5 (Lipinski et al. 1997). Panduratin A is almost appropriate for Lipinski’s rule of five, except for its high logP data, which implies low water solubility and leads to low oral bioavailability. One of the methods to improve solubility is the addition of cyclodextrin (Jain and Chella 2021); therefore, β-cyclodextrin was added to fingerroot extract in a 1:1 ratio to increase the solubility of panduratin A. Large animals like beagle dogs are used to determine drug formulation absorption, due to the beagle dog having a similar gastrointestinal morphology and physiology to humans (Kararli 1995). The pharmacokinetics of fingerroot extract has previously been studied in beagle dogs, but there is limited information on the oral bioavailability, possible metabolic pathway, and excretion recovery of panduratin A (Choi et al. 2020). Therefore, we conducted an experiment in beagle dogs to investigate the absolute oral bioavailability and disposition kinetics of panduratin A from a fingerroot extract formulation. The data obtained from this study may be useful for the phytopharmaceutical product development of fingerroot extract against SARS-CoV-2 infection in the near future.

## Materials and methods

### Chemicals

**Figure 1.**
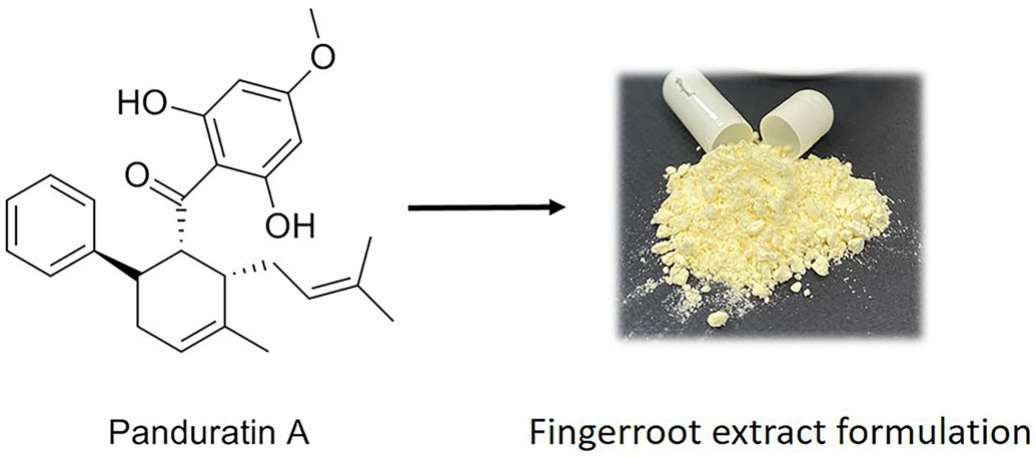
Chemical structure of panduratin A and the physical appearance of the fingerroot extract formulation.

The 500 mg fingerroot extract formulation capsules, containing 100 mg fingerroot extract, equivalent to 25 mg panduratin A, as determined by liquid chromatography tandem mass spectrometry (LCMS) analysis, and panduratin A (purity >93.6%) were provided by the Chao Phraya Abhaibhubejhr Hospital Foundation, Thailand (Figure 1). Dimethyl sulphoxide (DMSO) was purchased from Merck, Darmstadt, Germany. Panduratin A standard reference was purchased from Biosynth Carbosynth, UK, with ≥98% purity. Glycyrrhizin standard reference was purchased from Wako Pure Chemical Industries, Japan, with ≥90% purity.

### Fingerroot extract formulation

A 500 mg capsule of fingerroot extract formulation containing 100 mg fingerroot extract was developed (Chao Phraya Abhaibhubejhr Hospital Foundation, Thailand). β-Cyclodextrin (Wacker Chemie, Germany) was used as a complexing agent and stabilizer. In brief, the fingerroot extract was dissolved with 95% ethanol and then mixed with an aqueous solution of β-cyclodextrin (100 mg), which had been preheated until completely dissolved. The obtained mixture was then mechanically stirred at a constant 200 rpm at room temperature for 24 h. After that, the solvent was evaporated and dried in a hot air oven. The solid complex was ground into a powder using a grinding machine, and the powder was then dried in a hot air oven at 40 °C to determine the moisture content until it stabilized. Other diluents were added and mixed homogeneously, and the mixture was then packed into capsule shells. The fingerroot extract formulation was characterized and complied with the specification according to Table 1.

**Table 1.** The specification of fingerroot extract oral formulation.

Test	Specification	Test method	Result analysis
organoleptic:			
Physical characteristics	Yellow powder	Organoleptic evaluation	Conformed
Odour characteristics	Characteristic	Organoleptic evaluation	Conformed
Loss on drying (% w/w)	NMT 3.00	In-house	2.21
Disintegration (min)	Within 30 min	In-house	5.24
Panduratin A (% w/w)	–	LC-MS	5.07
Pinostrobin (% w/w)	–	LC-MS	12.29
Total aerobic microbial count (cfu/g)	<5 × 10^5^	In-house	<1
Total combined yeasts and moulds count (cfu/g)	<5 × 10^4^	In-house	<1
Bile-tolerant gram-negative bacteria (cfu/g)	<10^3^	In-house	<1
*Clostridium* spp. (cfu/g)	Absence	In-house	Absence
*Salmonella* spp. (cfu/10 g)	Absence	In-house	Absence
*Escherichia coli* (cfu/g)	Absence	In-house	Absence

### Animals

Twelve 18-month-old male beagles (weight 10 kg) were obtained from the Chulalongkorn University Laboratory Animal Centre (CULAC), Chulalongkorn University. The dogs were housed in a controlled environment with a temperature of 20–24 °C, relative humidity of 30–70%, and a 12 h light/dark cycle. Food was provided once a day, but water was available *ad libitum*. The dogs fasted for 12 h before the experiment, with free access to water. The dogs were randomly divided into three groups (one group for intravenous panduratin A and two groups for oral administration of the fingerroot capsule formulation). After administration of the test substances, the dogs were placed in metabolic cages for 72 h post-administration. The animal experiments were approved by the Institutional Animal Care and Use Committee of the CULAC, Chulalongkorn University, Bangkok, Thailand (Protocol number 2173035, Approval date: 20 September 2021) and were conducted in compliance with the ARRIVE guidelines.

### Pharmacokinetic experiment

For the intravenous panduratin A group, a single dose of 1 mg/kg panduratin A solution in DMSO was freshly prepared, then injected *via* the cephalic or saphenous vein of the dogs. For the two oral administration groups, single dose fingerroot capsules containing 5–10 mg/kg of panduratin A were orally administered to the dogs, then blood samples were collected until 72 h. After that, the dogs were orally administered the same dose of fingerroot capsules for seven consecutive days. Blood samples were collected from the cephalic or saphenous vein *via* a 22 G intravenous catheter on day 1 at 0 (pre-dose), 0.08, 0.25, 0.5, 1, 2, 4, 8, 24, 48 and 72 h and day 7 at 144 (pre-dose), 144.08, 144.25, 144.5, 145, 146, 148, 152, 168, 192 and 216 h after administration of the test substance, then placed into pre-heparinized blood collection tubes. The blood samples were centrifuged at 5000 *g* for 10 min at 4 °C to collect the plasma and then stored at −20 °C until analysis. Urine and faeces samples were collected in metabolic cages, 0–24 and 24–72 h after administration of the test substances. The volume of urine and the weight of faeces were recorded, and all samples were stored at −20 °C until analysis. To determine the health status of the dogs, before and after administration, blood samples were collected at 0 and 24 h after administration and sent to the Small Animal Hospital, Faculty of Veterinary Science, Chulalongkorn University for biochemical testing.

### Sample preparation and instrumentation

All biological samples (plasma, urine and faeces) were processed with protein precipitation to extract panduratin A from the samples: 200 µL of methanol with an internal standard (glycyrrhizin) was added to 50 µL of the biological sample and then vortex mixed for 10 min. Next, the sample was centrifuged at 12,000 *g* for 10 min at 4 °C, and 10 µL of supernatant was then injected into the LCMS instrument. Faeces samples were homogenized by adding methanol, then the mixture was centrifuged at 1500 *g* for 10 min at 4 °C to collect the supernatant. Where the concentration of a biological sample exceeded the linear calibration curve, blank matrices were used to dilute the sample prior to protein precipitation.

The LCMS system was operated using a LCMS-8060 (Shimadzu, Kyoto, Japan) equipped with a C18 reversed phase column, Synergi 4 µm Fusion-RP 80 Å 50 × 2 mm (Phenomenex, Torrance, CA). The column oven was maintained at 40 °C and operated using a mobile phase consisting of 0.2% formic acid in water (solvent A) and 100% methanol (solvent B), with a gradient system (0–0.5 min 10% B, 0.5–1.5 min increase to 90% B, 1.5–2.5 min 90% B, 2.5–3.5 min decrease to 10% B, 3.5–4.5 min 10% B), at a flow rate of 0.5 mL/min, with a 10 μL injection volume. The mass spectrometer was a triple quadrupole with negative mode electrospray ionization (ESI). The mass-to-charge ratios of panduratin A and glycyrrhizin were 405.10/165.90 and 821.25/350.90 Da, respectively. All the operations, acquisition and analysis of data were controlled by LabSolution software version 5.86 (Shimadzu, Japan). The LCMS method was validated for accuracy, precision, recovery and stability according to the guidance on bioanalytical method validation recommended by the FDA (USFDA 2018). The calibration curves of panduratin A were in the range of 10–1000 μg/L. The accuracy, precision, recovery and stability of the three quality control samples (10, 150 and 600 μg/L) obtained from the method were within an acceptable range (±15%), with a lower limit of quantification of 1.22 μg/L (Supplementary Figures S1, S2 and Tables S1-S3).

An Agilent 6540 QTOF (Agilent Technologies, Santa Clara, CA) equipped with Agilent HPLC 1260 (Agilent Technologies) was used to identify the major metabolites of panduratin A in biological samples; plasma, urine, and faeces of the beagle dogs after receiving intravenous panduratin A 1 mg/kg. A C18 reversed phase column, Synergi 4 µm Fusion-RP 80 Å 50 × 2 mm (Phenomenex), maintained at 35 °C was used as the stationary phase, and 0.1% formic acid in water (solvent A) and 0.1% formic acid in 50% acetonitrile/methanol (solvent B) were used as the mobile phase, with a gradient system (0 min 30% B, 0–20 min increase to 90% B, 20–25 min increase to 95% B, 25–35 min 95% B), at a flow rate of 0.5 mL/min, with a 10 μL injection volume. The LCMS with positive and negative mode ionization with ESI was conducted by screening a mass range from 100 to 1000 *m/z* at a scan rate of 4 spectra/sec. Mass Hunter software version B.06.00 (Agilent Technologies) was used for the qualitative analysis of mass spectra.

### Data analysis

PK solution software version 2.0 (Summit Research Services, USA) was used to analyse pharmacokinetic parameters by non-compartmental analysis. The pharmacokinetic parameters in this study were reported as follows: maximum plasma concentration (*C*_max_), time to reach maximum plasma concentration (*T*_max_), area under the plasma concentration-time curve from time 0–72 h (AUC_0–72_), area under the plasma concentration–time curve from time 0–infinity (AUC_0–inf_), absolute bioavailability, mean residence time (MRT), volume of distribution (*Vd*), total clearance (CL) and elimination half-life. Statistical analysis was performed in SPSS version 22.0 (IBM, Armonk, NY). The normality test was determined using the Shapiro–Wilk test. A paired Student’s *t*-test was used to determine significant differences in the health status of the dogs, and a paired Student’s *t*-test or Wilcoxon signed-rank test was used to determining significant differences in the pharmacokinetic parameters between oral groups. The percent recovery of panduratin A was calculated using the total amount of the test substance found in urine or faeces, divided by the given dose. All data are expressed as the mean ± standard deviation, except for the *T*_max_ and half-life, which are expressed as median (IQR). Differences were considered significant at *p* < 0.05.

## Results

### Animal tolerability

The health status of the dogs used in the study was determined by observing their physical appearance, monitoring weight and blood collection both pre- and post-administration of intravenous panduratin A or an oral formulation of fingerroot extract. None of the dogs showed a significant change in physical appearance or weight. The haematology, kidney and liver function values also showed no significant differences between pre-dose and post-dose in any group (Table 2). Continuous oral dosing of the fingerroot extract formulation for 7 d did not exert toxicity or abnormalities in beagle dogs.

**Table 2.** Physical and biochemical profiles of experimental beagles.

Biochemical parameters	Experimental groups					
	Baseline	Panduratin A 1 mg/kg i.v.	Fingerroot extract formulation (panduratin A 5 mg/kg) p.o. Day 1	Fingerroot extract formulation (panduratin A 5 mg/kg) p.o. Day 7	Fingerroot extract formulation (panduratin A 10 mg/kg) p.o. Day 1	Fingerroot extract formulation (panduratin A 10 mg/kg) p.o. Day 7
Physical appearance	Normal	Normal	Normal	Normal	Normal	Normal
Body weight	12.26 ± 1.30	12.30 ± 1.21	12.29 ± 0.89	12.46 ± 1.06	12.18 ± 1.26	12.28 ± 1.18
White blood cell (x 10^3^/µL)	8.13 ± 1.60	7.83 ± 1.09	8.38 ± 1.63	8.61 ± 1.30	8.50 ± 1.15	8.94 ± 0.81
Red blood cell (x 10^6^/µL)	6.16 ± 0.47	6.03 ± 0.66	6.55 ± 0.33	5.91 ± 0.53	6.59 ± 0.52	6.11 ± 0.42
Haemoglobin (g/dL)	14.4 ± 0.8	14.2 ± 1.3	15.5 ± 0.7	13.8 ± 1.2	14.9 ± 1.1	14.4 ± 1.00
Platelet (x 10^3^/µL)	233 ± 32	241 ± 44	233 ± 25	232 ± 33	268 ± 30	253 ± 32
Blood urea nitrogen (mg/dL)	13.6 ± 0.6	11.9 ± 1.6	12.7 ± 2.0	13.0 ± 2.0	11.1 ± 1.0	12.1 ± 1.6
Creatinine (mg/dL)	0.7 ± 0.1	0.6 ± 0.1	0.7 ± 0.1	0.8 ± 0.1	0.7 ± 0.1	0.7 ± 0.1
AST (U/L)	31 ± 6	24 ± 6	24 ± 6	29 ± 6	24 ± 4	25 ± 5
ALT (U/L)	42 ± 4	44 ± 7	46 ± 9	47 ± 8	46 ± 7	45 ± 6
Alkaline phosphatase (U/L)	39 ± 2	35 ± 4	32 ± 3	32 ± 7	29 ± 4	29 ± 4

AST: aspartate transaminase; ALT: alanine transaminase

Data are expressed as mean ± SD (*n* = 4). Decimal numbers were reported according to laboratory standard of Small Animal Hospital, Faculty of Veterinary Science, Chulalongkorn University.

### Plasma concentration-time profiles

**Figure 2.**
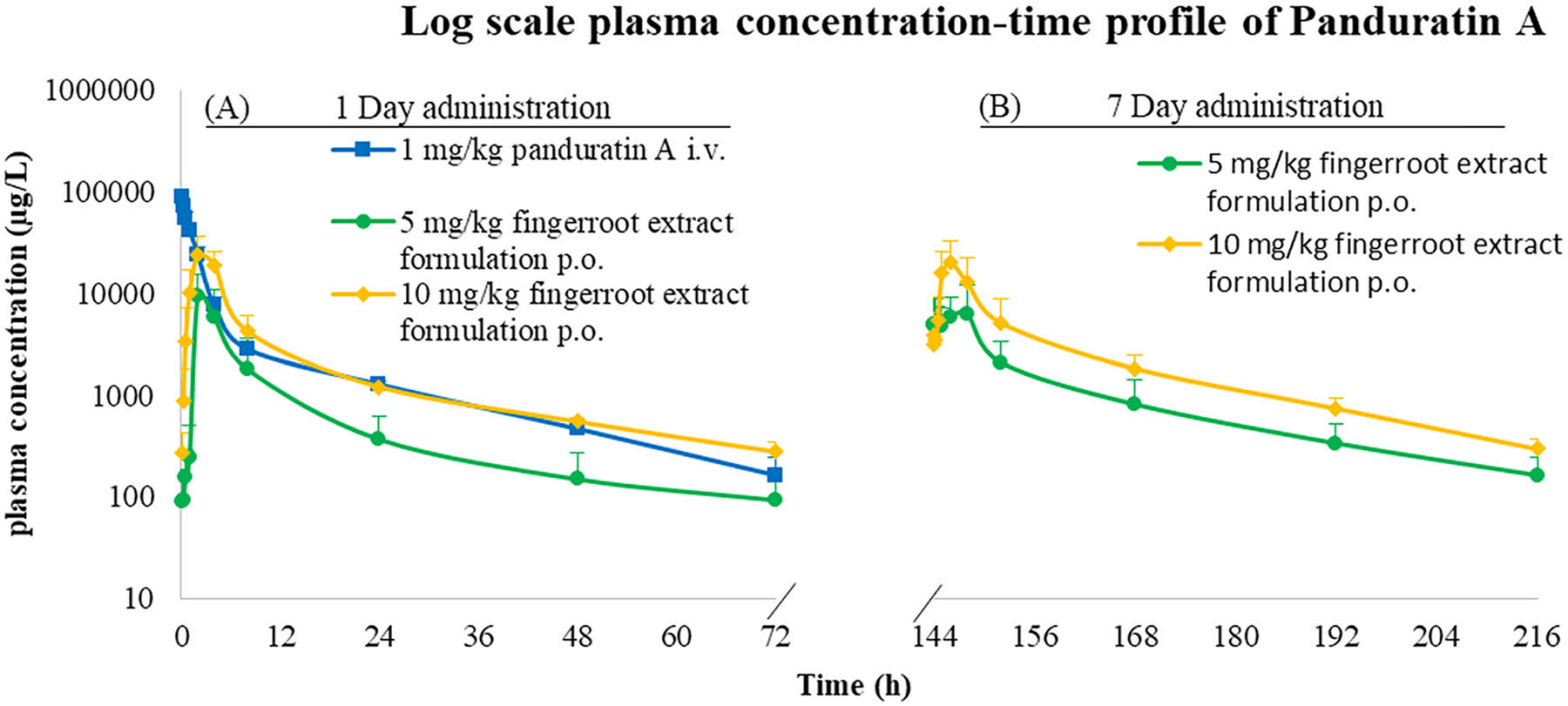
Plasma concentration-time profile of panduratin A after intravenous dosing as pure panduratin A 1 mg/kg, and oral dosing as fingerroot extract formulation containing panduratin A 5–10 mg/kg. (A) single administration on day 1. (B) multiple administration for seven consecutive days.

The plasma concentration-time profile of panduratin A after intravenous administration and oral administration of fingerroot extract formulation are shown in Figure 2. For the intravenous administration of panduratin A 1 mg/kg, the plasma concentration reached a maximum of approximately 100,000 µg/L, then declined to 100–200 µg/L after 72 h. A single oral administration of two capsules of the fingerroot extract formulation (equivalent to panduratin A 5 mg/kg) showed that the plasma concentration reached a maximum of approximately 10,000 µg/L, then declined to 100 µg/L after 72 h. Meanwhile, the maximal panduratin A plasma concentration of a single oral administration of four capsules of fingerroot extract formulation (equivalent to panduratin A 10 mg/kg) was approximately two-fold higher than the 5 mg/kg dose, demonstrating the tendency of dose proportionality of the panduratin A in the fingerroot extract oral formulation. After seven consecutive days of multiple oral administrations of the fingerroot extract formulation, the maximum plasma concentration of panduratin A was not significantly different from the first day of administration, indicating that panduratin A was not accumulated in the body of the dogs. The accumulation ratio of panduratin A was approximately 0.90–1.10 after continuous oral dosing of the fingerroot extract formulation for 7 d.

### Pharmacokinetic parameters

The pharmacokinetic parameters of panduratin A for all experimental groups are listed in Table 3. For the intravenous injection of panduratin A 1 mg/kg, the main pharmacokinetic parameters of AUC_0–72_, MRT, Vd, CL and half-life were 181,297 ± 27,374 µg.h/L, 23.90 ± 3.27 h, 0.1 ± 0.01 L/kg, 0.01 ± 0.01 L/h/kg and 15.25 h, respectively. For a single oral dose of fingerroot extract formulation equivalent to panduratin A 5 mg/kg, the main pharmacokinetic parameters of *C*_max_, *T*_max_, AUC_0–72_, MRT and half-life were 12,416 ± 2,326 µg/L, 2 h, 69,774 ± 36,907 µg.h/L, 41.05 ± 1.91 h and 11.38 h, respectively. The pharmacokinetic parameters after multiple oral administrations of fingerroot extract formulation equivalent to panduratin A 5 mg/kg exhibited no statistical difference compared to the single administration, with an accumulation ratio of 0.98. Increasing the dose of fingerroot extract formulation equivalent to panduratin A 10 mg/kg displayed significant differences in *C*_max_, AUC_0–72_ and AUC_0–inf_, which were approximately 2-fold compared with the 5 mg/kg dose. The absolute oral bioavailability of panduratin A in the fingerroot extract formulation was approximately 7–9%. The elimination half-life of panduratin A was approximately 10–15 h after intravenous or oral administration at 1–10 mg/kg/d.

**Table 3.** Pharmacokinetic parameters of panduratin A after intravenous injection of 1 mg/kg panduratin A, oral administration of fingerroot extract formulation equivalent to panduratin A 5–10 mg/kg as single and repeated doses.

Pharmacokinetic parameters	Panduratin A				
	Panduratin A 1 mg/kg i.v.	Fingerroot extract formulation (panduratin A 5 mg/kg) p.o. Day 1	Fingerroot extract formulation (panduratin A 5 mg/kg) p.o. Day 7	Fingerroot extract formulation (panduratin A 10 mg/kg) p.o. Day 1	Fingerroot extract formulation (panduratin A 10 mg/kg) p.o. Day 7
*C*_max_^a^ (µg/L)	115,487 ± 25,131	12,416 ± 2,326	9,981 ± 2,596	26,319 ± 8,221*	24,103 ± 3,428*
*T*_max_^b^ (h)	N/A	2.00 (0.50)	2.00 (1.50)	2.00 (0.50)	2.00 (0.25)
AUC_0–t_^a^ (µg.h/L)	181,297 ± 27,374	69,774 ± 36,907	68,500 ± 4,138	164,250 ± 48,166*	172,576 ± 32,465*
AUC_0–inf_^a^ (µg.h/L)	209,268 ± 18,929	73,711 ± 38,467	72,478 ± 5,652	176,597 ± 50,205*	178,682 ± 30,103*
Absolute bioavailability^c^ (%)	N/A	7.04	N/A	8.44	N/A
Accumulation ratio^d^	N/A	N/A	0.98	N/A	1.05
MRT^a^ (h)	23.90 ± 3.27	41.05 ± 1.91	41.70 ± 10.36	39.07 ± 9.27	37.53 ± 0.83
Vd^a^ (L/kg)	0.1 ± 0.01	2.20 ± 0.99	1.70 ± 0.50	2.08 ± 0.91	2.18 ± 1.86
CL^a^ (L/h/kg)	0.01 ± 0.01	0.08 ± 0.03	0.08 ± 0.05	0.06 ± 0.02	0.07 ± 0.05
Half-life^b^ (h)	15.25 (4.68)	11.38 (1.03)	12.03 (0.55)	11.65 (2.88)	10.00 (1.48)

^a^Data are expressed as mean ± *SD*; ^b^Data are expressed as median (IQR); ^c^Absolute bioavailability was calculated as (AUC_p.o._/DOSE_p.o._)/(AUC_i.v._/DOSE_i.v._) × 100; ^d^Accumulation ratio was calculated as AUC_0–t_ at day 7/AUC_0–t_ at day 1; *p < 0.05 for significant differences from fingerroot extract formulation 5 mg/kg. C_max_: maximum plasma concentration; *T*_max_: time to reach *C*_max_; AUC_0–t_: area under the plasma concentration–time curve from time 0–t h, *t* = 0–24 h in i.v. or 0–72 h in p.o.; AUC_0–inf_: area under the plasma concentration–time curve from time 0–infinity.

MRT: mean resident time; *Vd*: volume of distribution; CL: clearance.

### Biotransformation and excretion

**Figure 3.**
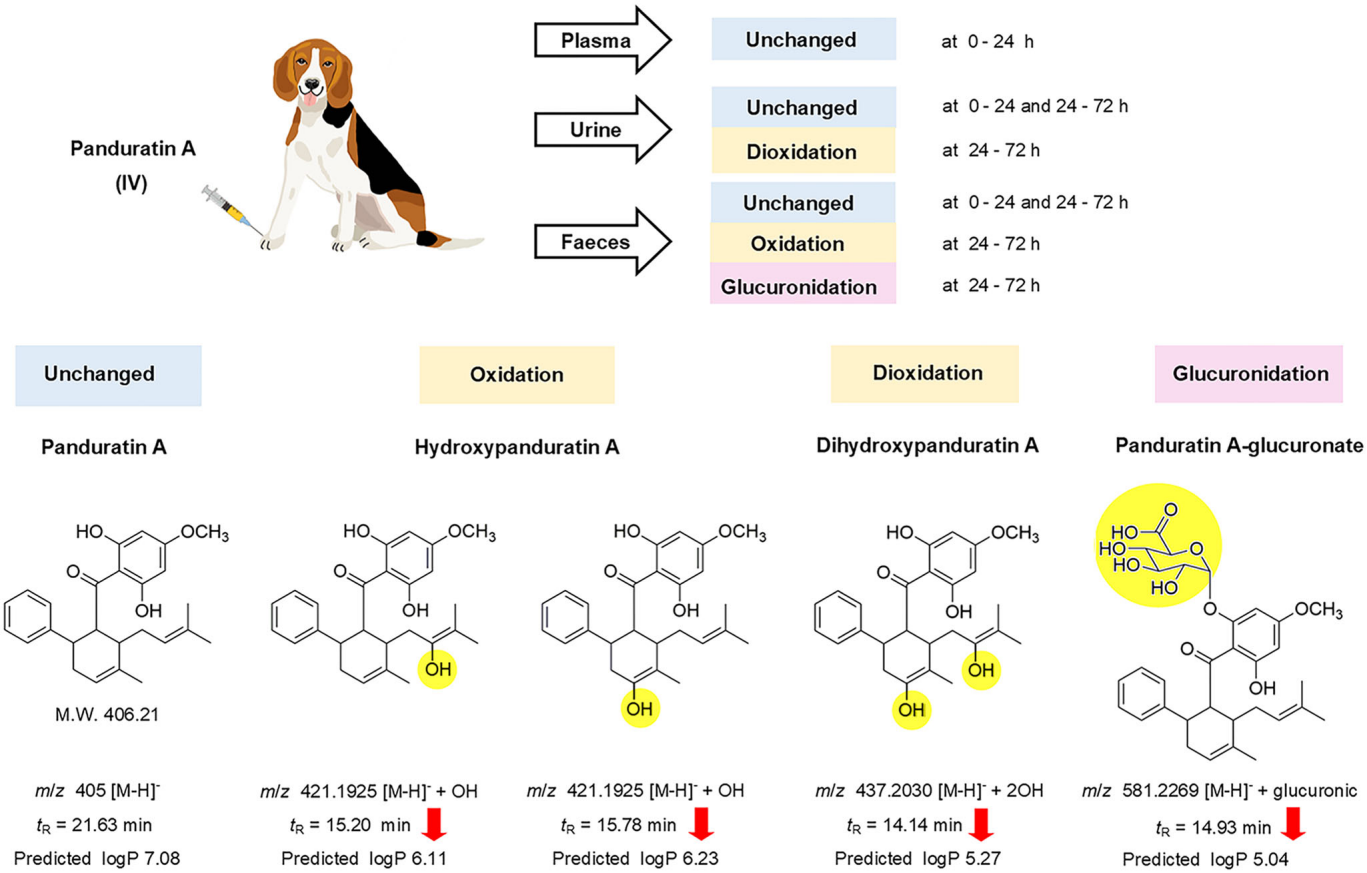
Proposed major metabolites, and excretion route of panduratin A in beagle dogs.

After intravenous administration of panduratin A, most of the compound was biotransformed before excretion, and only negligible amounts of unchanged panduratin A were excreted *via* the urine and faeces within 0–72 h (1–3% of the administered dose). Similarly, oral administration of the fingerroot extract formulation had approximately 1–10% of the administered dose as unchanged panduratin A in the excreta within 0–72 h (Table 4). The metabolites of panduratin A, which were identified by QTOF LCMS analysis, four compositions were found in urine and faeces, 24–72 h after intravenous administration of panduratin A (Figure 3). A major peak in the urine samples at 24–72 h was deduced to be a hydrogen adduct with dioxidation [M + H]^−^ + 2OH of panduratin A, at a molecular weight of 437.2030 g/mol with a retention time of 14.14 min. Meanwhile, two major peaks in the faeces samples at 24–72 h were identified as hydrogen adducts with oxidation [M + H]^−^ + OH of panduratin A; however, we deduced the molecules to be the same adduct with a different attachment of hydroxyl group by showing the identical molecular weight (421.1925 g/mol) but with different retention times (15.20 and 15.78 min). Moreover, a major peak in the faeces samples at 24–72 h was derived as a hydrogen adduct with glucuronic [M + H]^−^ + glucuronic of panduratin A, at a molecular weight of 581.2269 g/mol with a retention time of 14.93 min. Furthermore, predicted logP values of the proposed metabolites ([M + H]^−^ + 2OH, 2 [M + H]^−^ + OH, and [M + H]^−^ + glucuronic) were decreased, as 5.27, 6.11, 6.23 and 5.04, respectively, compared with panduratin A (7.08).

**Table 4.** The percentage recovery of panduratin A in excreta of all experimental groups.

Percent recovery	Experimental groups							
	Panduratin A 1 mg/kg i.v.		Fingerroot extract formulation (panduratin A 5 mg/kg) p.o. Day 1	Fingerroot extract formulation (panduratin A 5 mg/kg) p.o. Day 7		Fingerroot extract formulation (panduratin A 10 mg/kg) p.o. Day 1	Fingerroot extract formulation (panduratin A 10 mg/kg) p.o. Day 7	
	0–24 h	24–72 h	0–24 h	0–24 h	24–72 h	0–24 h	0–24 h	24–72 h
Urine								
Panduratin A	0.28 ± 0.21	0.89 ± 0.74	0.02 ± 0.01	0.72 ± 0.53	2.00 ± 2.04	0.16 ± 0.21	0.65 ± 0.98	0.64 ± 1.03
Faeces								
Panduratin A	2.51 ± 1.28	0.82 ± 0.49	7.35 ± 6.06	9.29 ± 5.90	3.19 ± 2.94	6.60 ± 3.70	10.81 ± 7.67	0.22 ± 0.13

Data are expressed as mean ± *SD* (*n* = 4).

## Discussion

Fingerroot extract and panduratin A have demonstrated the potential to reduce viral output and lung lesions in a SARS-CoV-2 *in vitro* study and hamster model, respectively (Kanjanasirirat et al. 2020). The drug development process requires data from preclinical studies to establish clinical trials (Reichel and Lienau 2016). In this study, the pharmacokinetic parameters of panduratin A as a pure compound and in a fingerroot extract formulation were evaluated in a dog pharmacokinetic model. All of the experimental animals tolerated the test substances well after intravenous or oral administration and showed no signs of toxicity. The results indicated minimal toxicity of the test substances in beagle dogs within the dosing interval of the test compounds. Our findings are consistent with toxicity studies of other groups who fed 60–240 mg/kg fingerroot extract to rats for 60 d and found no significant changes in weight, blood and biochemical parameters or in liver and kidney functions and histopathology (Saraithong et al. 2010). In addition, 350 mg of fingerroot extract was given to functional dyspepsia patients three times daily for 4 weeks and found to be well tolerated, with no change in gastric histology (Chitapanarux et al. 2021). Based on this toxicity data, fingerroot extract seems to be safe for use in animals and humans.

The maximal plasma-concentration of panduratin A from the oral administration of fingerroot extract formulation equivalent to panduratin A 5–10 mg/kg was shown to exceed the IC_50_ value of panduratin A in the SARS-CoV-2 *in vitro* study by approximately 25 times, while the minimal plasma-concentration at 24 h remained approximately 1–3 times higher than the IC_50_ value (Kanjanasirirat et al. 2020). The absolute oral bioavailability of panduratin A in the fingerroot extract formulation was approximately 7–9%, and increasing the dose of the fingerroot extract formulation showed no significant difference in absolute bioavailability. The two-fold increase of *C*_max_, AUC_0–72_ and AUC_0–inf_ was proportional to the increase in dose, which might indicate a linear dose–response relationship of panduratin A. In another study, the pharmacokinetic profiles of panduratin A in beagle dogs were slightly different in *T*_max_, and CL parameters (Choi et al. 2020). The difference in pharmacokinetic parameters might be the result of different extraction methods for the fingerroot, as CO_2_ extraction was used in our method, while ethanol extraction was used in the study by Choi et al. (2020). The different extraction techniques could affect the quantity of the active compound and the variety of minor compositions, which might result in a change in the pharmacokinetic profiles (Sun et al. 2019). In addition, Choi et al. (2020) used a corn oil suspension, while we used a cyclodextrin formulation packed into capsules, and the inactive ingredients and different formulations also play a crucial role in improving absorption (Huang et al. 2015). Herbal medicines normally have low water solubility and high lipophilic properties, which cause insufficient absorption and bioavailability, and the addition of excipients is a way to improve water solubility and oral bioavailability (Kesarwani et al. 2013). Cyclodextrin, a complex of cyclic oligosaccharides, is used as an excipient to enhance the water solubility and bioavailability of drugs or herbal medicines and is safe for use in animals and humans (EMA 2017). Cyclodextrin is also reported to be a water solubility and bioavailability enhancer in *Kaempferia parviflora* Wall. ex Baker extract and zerumbone isolated from *Zingiber zerumbet* (L.) Roscoe ex Sm., which are both plants of the family Zingiberaceae, similar to fingerroot (Mekjaruskul et al. 2013; Muhammad Nadzri et al. 2013). By improving the water solubility, the active compounds can be better absorbed, resulting in increased plasma levels and oral bioavailability.

The half-life values of panduratin A were approximately 10–15 h in both intravenous panduratin A and the oral fingerroot extract formulation, which could indicate long systemic exposure. With the high lipophilicity predicted by the partition coefficient of panduratin A (PubChem 2004), the molecule prefers to reside in the tissue compartments, rather than circulate in the bloodstream. This finding is related to a study of the oral administration of fingerroot extract in rats, which demonstrated the distribution of panduratin A to various tissues, including skin, lung, heart, gum, liver, spleen, kidney and brain (Won et al. 2021). Panduratin A was mostly biotransformed after intravenous or oral administration of the test substances and then slowly excreted as biotransformed products from 24 to 72 h. The QTOF LCMS analysis results proposed the metabolites of panduratin A in the excreta, which decreased the predicted logP and reduced the lipophilicity of panduratin A. In faeces at 24–72 h, a metabolite of panduratin A with glucuronidation was found, which was the lowest XlogP metabolite. Normally, glucuronide metabolite of drugs excretes to the intestine through biliary excretion then is hydrolysed by the gut microbiome and reabsorbed into the systemic circulation (Zhou et al. 2019). The glucuronide metabolite of panduratin A found in faeces is likely from high lipophilicity. Gossypol, a natural phenol derived from the cotton plant with high lipophilicity (XlogP = 6.9), is also found to excrete glucuronide metabolite in faeces (Liu et al. 2014). The possible biotransformation of panduratin A consists of phase I (oxidation) and phase II (glucuronidation) metabolism, which increases the water solubility of panduratin A, resulting in an escalated elimination of the substance (Garza et al. 2022). Further study is necessary to identify and quantify the major metabolites of panduratin A for the development of a fingerroot extract product.

## Conclusion

Panduratin A was shown to be safe in beagle dogs as an intravenous pure compound and a fingerroot extract oral formulation. An increased dose of the fingerroot extract could increase the systemic exposure of panduratin A in a dose-proportional manner. The majority of panduratin A was biotransformed to several products *via* oxidation and glucuronidation, and mainly excreted *via* the faecal route. The pharmacokinetic results could assist in the development of phytopharmaceuticals from fingerroot in the near future.

## Supplementary Material

Supplemental Material

## Data Availability

The data that support the findings of this study are available from the corresponding author upon reasonable request. Some data may not be made available because of privacy or ethical restrictions.
