# Supplementary material for: Absolute oral bioavailability and possible metabolic pathway of panduratin A from Boesenbergia rotunda extract in beagle dogs
Source: Pharm Biol. 2023 Mar 30;61(1):590–7. doi: 10.1080/13880209.2023.2190777 (PMC10064817; doi:10.1080/13880209.2023.2190777)
Supplement: Supplemental Material [file IPHB_A_2190777_SM6798.docx]

**Absolute oral bioavailability and possible metabolic pathway of panduratin A from *Boesenbergia rotunda* extract in Beagle dogs**

Tussapon Boonyarattanasoonthorn^a#^, Teetat Kongratanapasert^b#^, Apisada Jiso^b^, Pinnakarn Techapichetvanich^b^, Nitra Nuengchamnong^c^, Kittitach Supannapan^d^, Anusak Kijtawornrat^a^, Phisit Khemawoot^b^

*^a^ Department of Physiology, Faculty of Veterinary Science, Chulalongkorn University, Bangkok, Thailand*

*^b^ Chakri Naruebodindra Medical Institute, Faculty of Medicine Ramathibodi Hospital, Mahidol University, Samutprakarn, Thailand*

*^c^ Science Laboratory Center, Faculty of Science, Naresuan University, Phitsanulok, Thailand*

*^d^ Chao Phraya Abhaibhubejhr Hospital Foundation, Prachinburi, Thailand*

***^#^*** *The work has been contributed by these authors equally.*

**^*^Corresponding authors**

Phisit Khemawoot, Ph.D.

Chakri Naruebodindra Medical Institute, Faculty of Medicine Ramathibodi Hospital, Mahidol University, Bang Phli, Samut Prakarn 10540, Thailand

E-mail address: phisit.khe@mahidol.ac.th

**Contents**

**Supplementary figure S1. LCMS chromatograms.**

(A) fingerroot extract from QTOF LCMS analysis.

(B) panduratin A from triple quadrupole LCMS analysis.

(C) blank plasma from triple quadrupole LCMS analysis.

(D) blank plasma spiked with LLOQ from triple quadrupole LCMS analysis.

(E) a sample from triple quadrupole LCMS analysis.

**Supplementary figure S2. LCMS mass spectra.**

(A) panduratin A from QTOF LCMS analysis.

(B) glycyrrhizin from QTOF LCMS analysis.

**Supplementary table S1. Accuracy & Precision of panduratin A.**

**Supplementary table S2. Stability of panduratin A.**

**Supplementary table S3. Recovery of panduratin A.**


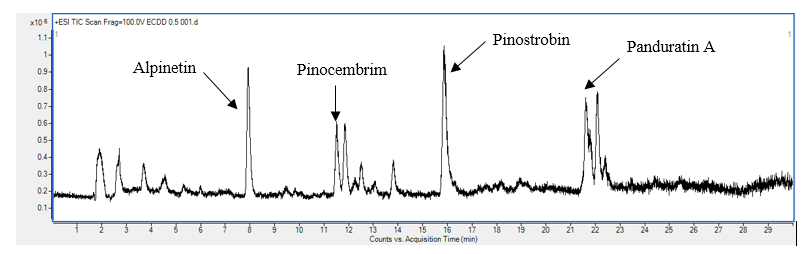


**A**


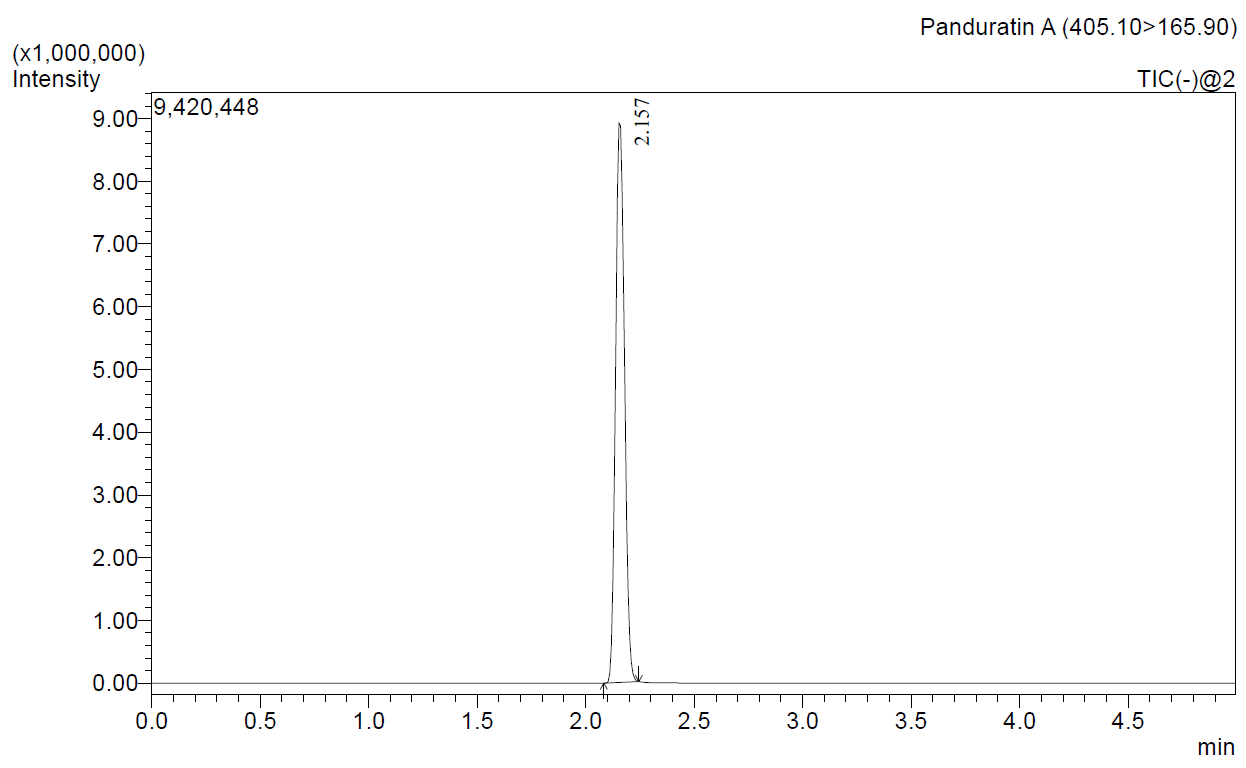


**B**


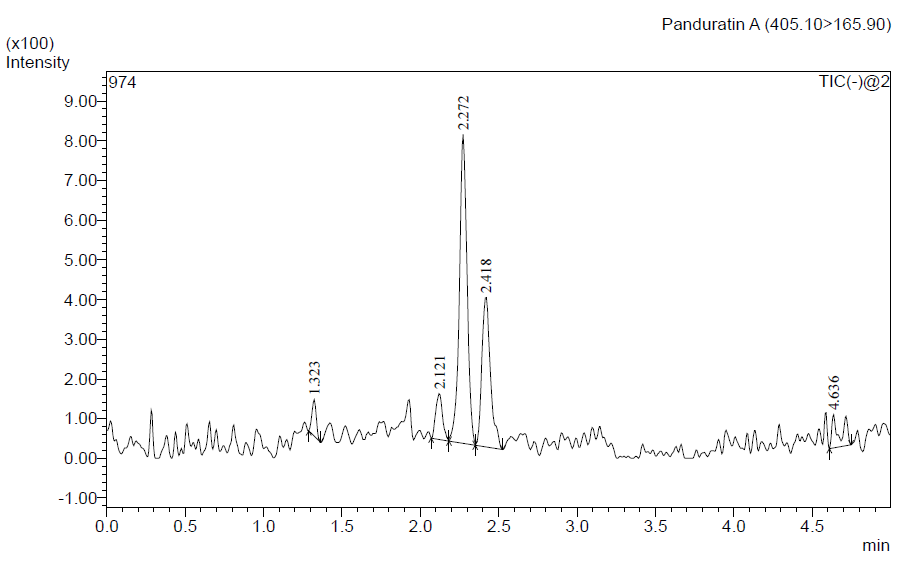


**C**


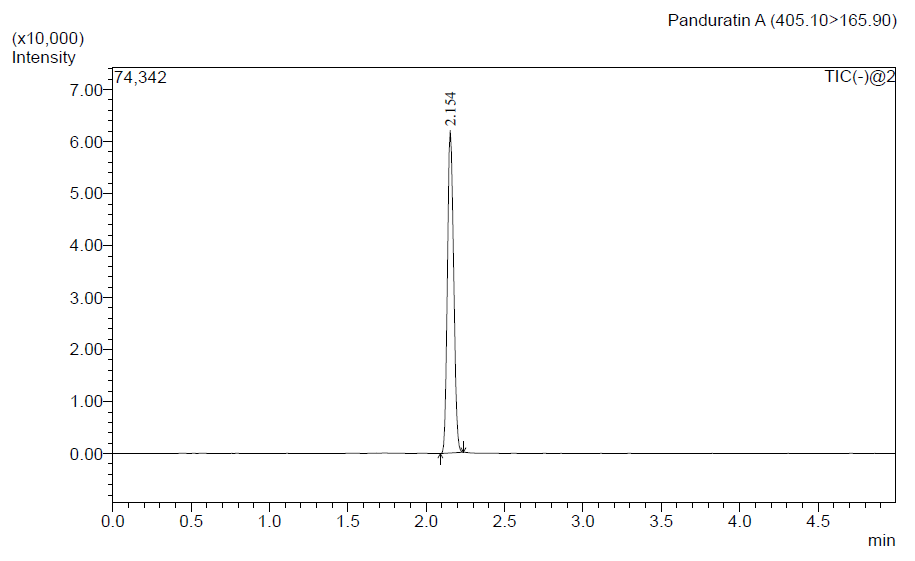


**D**


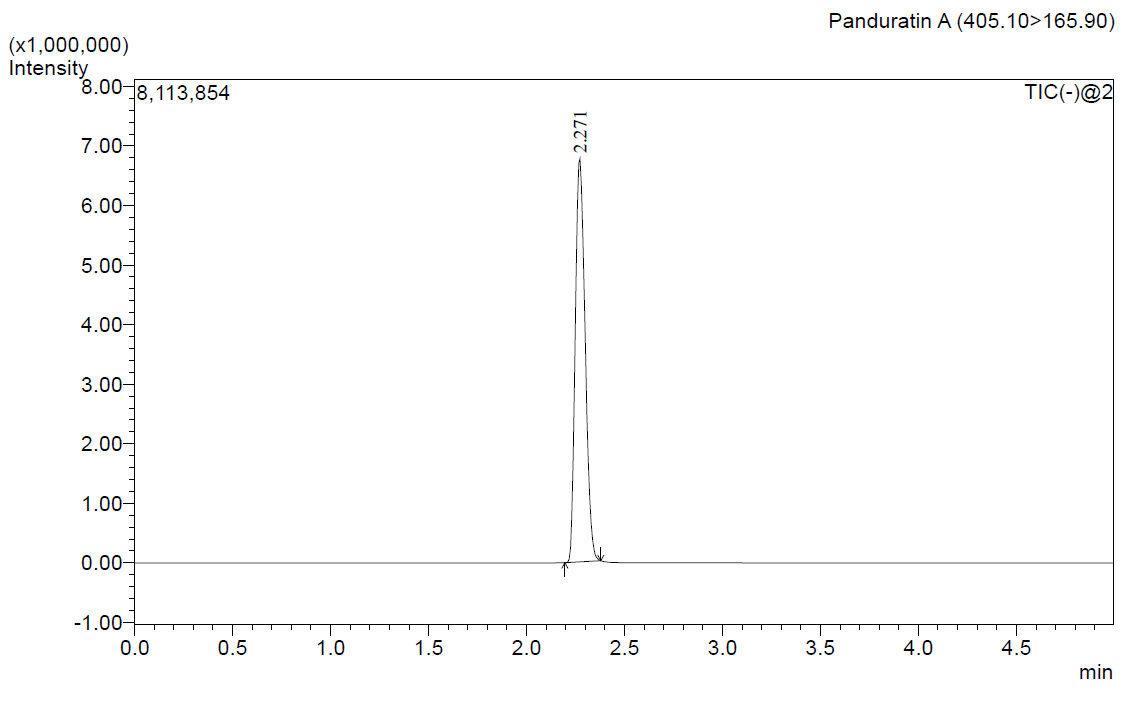


**E**

Supplementary figure S1. LCMS chromatograms. (A) fingerroot extract from QTOF LCMS analysis. (B) panduratin A from triple quadrupole LCMS analysis. (C) blank plasma from triple quadrupole LCMS analysis. (D) blank plasma spiked with LLOQ from triple quadrupole LCMS analysis. (E) a sample from triple quadrupole LCMS analysis.

**A**

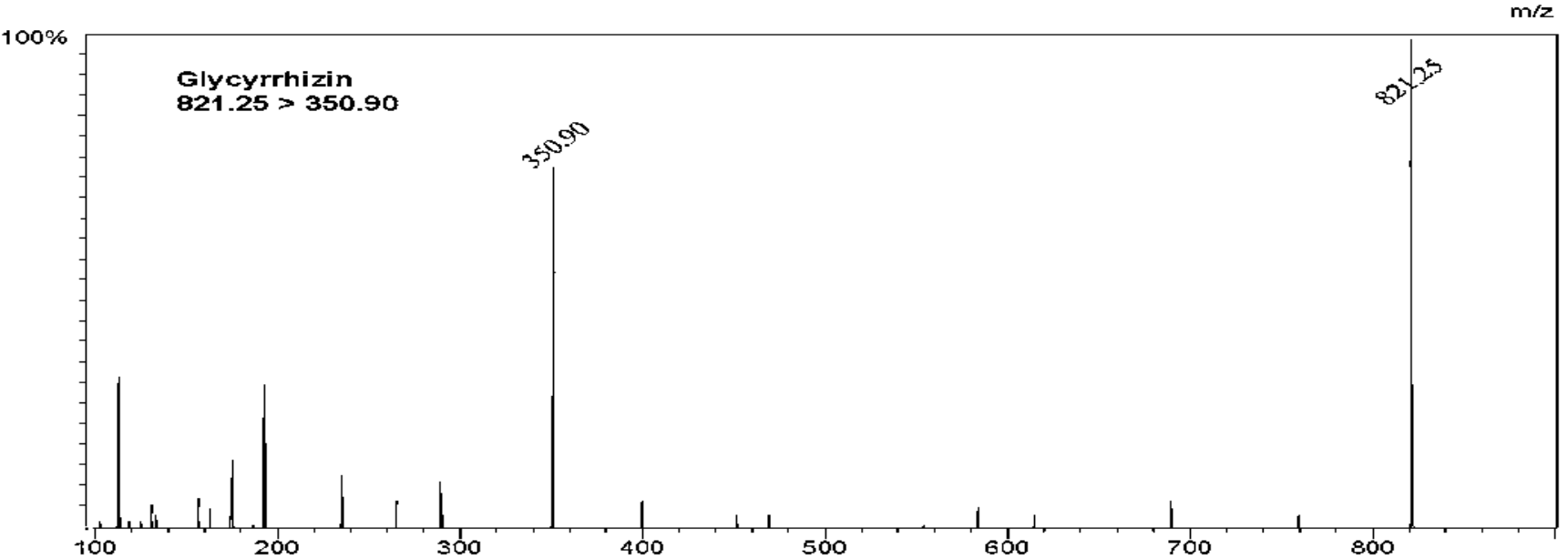


**B**

Supplementary figure S2. LCMS mass spectra (A) panduratin A from QTOF LCMS analysis. (B) glycyrrhizin from QTOF LCMS analysis.

Supplementary table S1. Accuracy & Precision of panduratin A.

| Concentration (µg/L) | | Panduratin A | |
| --- | --- | --- | --- |
|  |  | Accuracy (% RE) | Precision (% CV) |
| Intra-day | | | |
| High | 600 | 3.45 | 8.47 |
| Medium | 150 | 8.17 | 8.65 |
| Low | 10 | 13.02 | 9.02 |
| Inter-day | | | |
| High | 600 | 4.90 | 9.72 |
| Medium | 150 | 9.50 | 10.65 |
| Low | 10 | 14.51 | 7.10 |

RE, relative error; CV, coefficient of variation.

Supplementary table S2. Stability of panduratin A.

|  | Storage conditions | Concentration  (µg/L) | Mean | SD | Accuracy (%RE) | Precision (%CV) |
| --- | --- | --- | --- | --- | --- | --- |
| Panduratin A | room temperature (12 h) | 600 | 628.57 | 18.97 | 4.76 | 3.02 |
|  |  | 150 | 156.72 | 5.11 | 4.48 | 3.26 |
|  |  | 10 | 10.98 | 0.93 | 9.75 | 8.47 |
|  | 3 freeze-thaw cycles | 600 | 626.42 | 21.25 | 4.40 | 3.39 |
|  |  | 150 | 160.47 | 1.41 | 6.98 | 0.88 |
|  |  | 10 | 10.31 | 0.63 | 3.08 | 6.08 |
|  | storage at  -20°C | 600 | 580.72 | 35.16 | -3.21 | 6.05 |
|  |  | 150 | 152.78 | 17.30 | 1.86 | 11.33 |
|  |  | 10 | 10.76 | 1.57 | 7.58 | 14.55 |
|  | autosampler | 600 | 578.83 | 32.02 | -3.53 | 5.53 |
|  |  | 150 | 154.37 | 7.85 | 2.91 | 5.09 |
|  |  | 10 | 10.32 | 0.98 | 3.25 | 9.44 |

RE, relative error; CV, coefficient of variation.

Supplementary table S3. Recovery of panduratin A.

|  | Concentration (µg/L) | | Mean | SD | Absolute recovery (%) |
| --- | --- | --- | --- | --- | --- |
| Panduratin A | UQC 600 µg/L | Extract | 604.02 | 23.80 | 92.28 |
|  |  | Un-extract | 654.58 | 21.59 |  |
|  | MQC 150 µg/L | Extract | 158.92 | 8.26 | 98.73 |
|  |  | Un-extract | 160.97 | 7.41 |  |
|  | LQC 10 µg/L | Extract | 11.30 | 1.05 | 104.07 |
|  |  | Un-extract | 10.86 | 1.35 |  |
